# Endogenous Sphingolipid Signaling Pathway Implicated in the Action of *Croton membranaceus* on the Prostate Gland in BPH Patients

**DOI:** 10.3390/medicines4040084

**Published:** 2017-11-18

**Authors:** George Awuku Asare, Yvonne Anang, Daniel K. Afriyie, Brodrick Yeboah Amoah, Bernice Asiedu, Derek Doku, Hannah Serwah Ocansey, Nana Yaw Odei Danso, Prince Tekpor, Sarah Osam

**Affiliations:** 1 Chemical Pathology Unit, Department of Medical Laboratory Sciences, School of Biomedical and Allied Health Sciences, College of Health Sciences, University of Ghana, P.O. Box KB 143, Korle-bu, Accra, Ghana; vongh2002@yahoo.co.uk (Y.A.); abybrodrick@yahoo.com (B.Y.A.); benysea2@gmail.com (B.A.); amarteydd@gmail.com (D.D.); ohannahsaige1@yahoo.com (H.S.O.); odynaan@gmail.com (N.Y.O.D); ptekpor@yahoo.com (P.T); sarahosam95@gmail.com (S.O.); 2 Department of Pharmacy, Ghana Police Hospital, Cantonments, Accra, Ghana; dspdan77@yahoo.com

**Keywords:** Ceramide, sphingolipid, prostate, *Croton membranaceus*

## Abstract

**Background:** Croton membranaceus extract has apoptotic effects on BPH-1 cells. This study determined if the apoptotic effects were created through the ceramide pathway. **Methods:** The study was a follow-up to a previous observational study of 30 histologically confirmed patients with benign prostatic hyperplasia (BPH) who were on *C. membranaceus* ethanolic extract at 20 mg t.i.d orally for 3 mo. Thereafter, total and free prostate-specific antigen (PSA), lipid profile plus Apo lipoprotein A and B, ceramide/Sphingophospho-kinase 1 (SphK1) and 2 (SphK2), sphingosine lyase (SPL), the cytotoxic adducts of oxidative stress 4-hydroxy-2-nonenal (4HNE) and malondialdehyde (MDA), were determined. **Results:** Total and free PSA were significantly (*p* < 0.05) different after treatment. Apo lipoprotein A was significantly different (*p* = 0.024). The SphK1/SphK2 ratio reduced significantly (*p* = 0.049). Furthermore, SPL, ceramide, and MDA increased significantly after treatment (*p* = 0.05, *p* = 0.004, and *p* = 0.007, respectively). A weak positive correlation was found between high-density lipoprotein (HDL) cholesterol and SphK1, and HDL and ceramide before treatment (*p* = 0.036, r = 0.3826; *p* = 0.018, r = 0.4286, respectively. **Conclusions:** *C. membranaceus* uses the ceramide pathway by modulating the SphK1/SphK2 ratio and increasing SPL to generate oxidative stress and consequently apoptosis.

## 1. Introduction

Cell survival or death is regulated by a sphingolipid pathway that results in the growth of tumors [1]. Ceramide, a C18 lipid also known as a tumor suppressor lipid, and sphingosine are pro-apoptotic in nature, whereas the metabolic product sphingosine-1-phosphate (SIP) is pro-proliferative in nature [2]. These sphingolipids play a signaling role in tumorigenesis [3]. Under pathogenic conditions, the conversion of ceramide to SIP is the primary mechanism leading to cell growth and proliferation. Thus, the pro-apoptotic and pro-proliferative sphingolipids are called the sphingolipid rheostat. The ratio of sphingosine or ceramide to SIP is a determinant of cell growth or death [4]. A high ratio favors apoptosis and the reverse favor cell proliferation.

Some studies have shown that ceramide exerts its apoptotic effect using the intrinsic pathway. In response to apoptotic stimuli, ceramide acts on a class of proteins known as Bcl-2 proteins, enhancing the activation of pro-apoptotic Bcl-2 proteins and inhibiting anti-apoptotic Bcl-2 proteins [5]. Ceramide also triggers mitochondrial outer membrane permeabilization (MOMP), activates the release of cytochrome C and other apoptogenic proteins into the cytosol, and initiates the production of reactive oxygen species, which enhance apoptosis [6].

The fate of a cell is also determined by ceramide and ceramide-1-phosphate. Growth inhibition and apoptotic activities occur in the long chain bases as well as in ceramide, where their phosphorlylated metabolites stimulate proliferation and growth. For normal cell function, the modulation of these lipids and their opposing metabolites is therefore essential [7,8]. The catalytic actions of sphingosine kinases on sphingosine biosynthesis result in the production of long-chain bases [9]. Cellular uptake mechanisms result in phosphorylation at position one of these long-chain bases. Furthermore, dephosphorylation is possible through the action of sphingosine-1-phosphate phosphatases or type 2 phosphatidate phosphohydrolases [10,11]. On the other hand, sphingosine phosphate lyase (SPL) metabolizes the phosphate intermediates irreversibly into long-chain phosphoethanolamines and aldehydes [12].

Sphingosine kinase has two isoenzymes, sphingosine phosphokinase 1 (SphK1) and sphingosine phosphokinase 2 (SphK2), located in different subcellular compartments [13]. SphK1 is found in the cytosol and transferred into the plasma membrane when stimulated. Cell proliferation has been shown to be enhanced by increased SphK1 expression in cell lines with the promotion of G1-S transition, which was previously protected from apoptosis. However, S1P has been suggested to suppress apoptosis as a result of SphK1 induction not being mediated by S1P receptors [14,15]. An increased SphK1 expression has been observed in solid tumors and in cancers, including breast, colon, rectum, and ovary cancers [16,17].

The combination of SPL-enforced expression with SphK1 may be a silencing strategy that decreases S1P content in the prostate, which may result in increasing the sensitivity of prostate cancer cells to anticancer therapies [18].

On the other hand, SphK2 is said to have a BH3-only motif and functions upon stimuli from the cytosol to the nucleus, promoting apoptosis through the mitochondrial pathway. In several cell lines, SphK2 expression has been associated with the inhibition of DNA synthesis [19]. SphK2 and SphK1 use SIP as a common substrate; however, SphK2-induced apoptosis is independent of the activation of S1P receptors, but interacts with the anti-apoptotic member Bclx-L [20].

The downregulation of SphK2 reduces the conversion of sphingosine to ceramide in the salvage pathway, whereas downregulation of SphK1 enhances this process, resulting in elevated ceramide levels [21]. Thus, it has been suggested that SphK1 and SphK2 have opposite effects.

In contrast, downregulation of critical oncogenes by a selective SphK2 inhibitor hindered prostate cancer progression [22]. SphK2 has been shown to be capable of promoting proliferation in breast cancer cells [23].Therefore, the role of these two enzymes is not clearly understood and may vary in different pathological settings.

Several phytotherapeutic agents are purported to have success in the management of benign prostatic hyperplasia (BPH). Two monoherbal plants, *Urticadioica* L. and *Trifolium pretense* (red clover), have been shown to reduce BPH [24]. *Croton membranaceus* was also observed to shrink the prostate in BPH patients, and an in vivo study implicated a possible apoptotic mechanism [25]. However, almost no research has been able to show the exact mechanism by which these plant medicines work in humans. The aim of this study was to determine if the apoptotic nature of the extract of *C. membranaceus* occurs through the ceramide pathway.

## 2. Materials and Methods

### 2.1. Study Design and Site

The study was a retrospective study, using archival samples from an observational study in patients who opted for *C. membranaceus* use in the management of their BPH, at the Ghana Police Hospital. The Ghana Police Hospital is one of the 11 centers in the country approved by the Ministry of Health to dispense plant medicine to patients who opt for that type of treatment. Blood samples of 30 patients who used the ethanolic root extract of *C. membranaceus* capsules (20 mg t.i.d.) for a period of 3 months were analyzed. Frozen archival blood samples were selected and analyzed. Before treatment and after treatment samples were used. The characterized phtyochemicals in *C. membranaceus* were terpenoids, dipeteroids, furano-clerodanediterpeniod, crotomembranafuran, *N*[*N*-(2-methylbutanoyl) glutaminoyl-2-phenylethylamine, glutarimide alkaloid, julocrotine, beta-sitosterol, beta-sitosterol-3-d-glucoside, labdanediterpioid, gomojoside H, and dl-thrietol [26].

### 2.2. Ethical Issues

The Ethics and Protocol Review Committee of the School of Biomedical and Allied Health Sciences granted approval for the study with ethics number SAHS-ET/SAHS/PSM/ML/09/AA/26A/2012-2013. The Ghana Police Hospital administration granted approval for the study to be undertaken at the facility. Participants whose data and samples were used provided informed consent. The study complied with the Helsinki Declaration of 1964, with revision in October 2008.

### 2.3. Methods

Biochemical assays, including total and free prostate specific antigen (TPSA and FPSA), were performed using AccuBind total and free PSA ELISA kits purchased from MonoBind Inc. (Lake Forest, CA, USA). The ELISA plate was coated with highly specific mono-clonal anti-PSA antibodies, and assays were performed according to the manufacturer’s instructions.

The lipid profile, including total cholesterol (TC), triglycerides (TG), high density lipoprotein (HDL), low density (LDL) apolioprotein A-1 (Apo A), and apolioprotein B (Apo B), were determined using BioSystem A25 (Madrid, Spain) with BioSystems reagents.

Sphingosine phosphokinase 1 and 2 ELISA kits were purchased from MyBiosource (San Diego, CA, USA). Ceramide, sphingosine-1-phospahe lyase and malondialdehyde (MDA) ELISA kits were purchased from Sunlong Biotech Co. Ltd. (Hangzhou, China). All tests were performed using assay protocols as directed by the manufacturers.

### 2.4. Statistical Analysis

IBM SPSS statistics for Windows, Version 21.0 (IBM Corporation, Armonk, NY, USA) and Microsoft Excel 2013 were used for the statistical analysis. Data are presented as mean ± standard deviation. Statistical analysis between groups was determined by student’s *t*-test. Correlation analysis was performed with Pearson correlation test. The level of significance was *p* < 0.050.

## 3. Results

Significant reductions were seen in total PSA (TPSA) and free PSA (TPSA) (*p* = 0.000 and *p* = 0.018, respectively) at the end of the treatment period (Table 1). From Table 2, a significant increase was observed in Apo A-1 after treatment (*p* = 0.024). Although SphK1 and SphK2 were lowered after treatment, differences were not significant. However, the SphK1:SphK2 ratios decreased significantly after treatment (*p* = 0.049). On the contrary, SPL increased significantly after treatment (*p* = 0.05) (Table 3). Ceramide increased from 64.77 ± 11.09 to 72.92 ± 10.83 ng/mL. The increase was highly significant (*p* = 0.004) (Table 4). Positive correlations were observed between HDL before treatment for SphK1 and ceramide (r = 0.382, *p* = 0.036; and r = 0.428, *p* = 0.018, respectively) (Table 5), and between TG and SPL (*p* = 0.015, r = 0.4370) before treatment (Table 6).

## 4. Discussion

The use of medicinal plants for various conditions is increasing globally. For 70–80% of most populations, these plants are most readily available and affordable. However, even in settings where allopathic medicine is widely available, some people have a deliberate preference for medicinal plants because they are thought to be natural and safe. In Italy, 50% of the BPH medications are phytotherapies, and in some European countries, first-line BPH treatment involves the use of phytotherapies [27].

A meta-analysis by Azimi et al. [24] revealed that, although many medicinal plants have been suggested to manage BPH given the results of in vitro experiments, few have demonstrated the same effects in vivo. Furthermore, the few in vivo experiments have not surpassed a few clinical markers, such as International Prostate Symptoms Score (IPSS) and Lower Urinary Tract Symptoms (LUTS). Additionally, a great dearth of information exists about the availability of the exact mechanism of action of the few that have been shown to be clinically successful.

*Croton membranaceus* has been used in Ghana over the past four decades for the management of BPH and has been shown to shrink the prostate in animal and human experiments [28,29]. Some pathways have been suggested for its action, including the dihydrotestosterone (DHT) reduction by the 5-α-dehydrogenase hormonal pathway, and possible antioxidant activities [30]. In vitro experiments have demonstrated that the aqueous plant extract is cytotoxic and genotoxic [31]. Another experiment at the molecular level showed that the antiproliferative effect on BPH-1 cells was due to a mitochondrion-dependent pathway leading to apoptosis [25]. Although the apoptotic mechanism has been accepted as a mode of action for the plant extract, the exact pathway has not been determined. In this study, a more convincing pathway is presented based on analysis of human serum samples obtained from a previous observational study.

The involvement of lipid metabolism in the pathogenesis of BPH has been demonstrated [32,33]. Metabolic syndrome (MetS) has been implicated in the etiology of BPH. In a related study, the frequent co-existence of MetS and BPH was confirmed [34]. This association seems to be a consequence of the MetS-related metabolic derangements, reduced sex hormone binding globulin (SHBG) levels, and changes in the sex hormones. In that study, the strongest significant predictor of BPH was metabolic syndrome. HDL was negatively correlated with inflammatory infiltrate grade and glandular disruption. Furthermore, after prostatectomy, prostate specimen weight was negatively correlated with HDL levels. Receiver operating characteristic (ROC) curves showed that inflammatory infiltrate grade, extent, and the presence of glandular disruption predicted reduced HDL-cholesterol with an accuracy of 67.4%, 72.4%, and 70.2%, respectively [35].

HDL has been found to be lower in BPH patients [36]. BPH has been suggested as a new metabolic disease in aging men that is correlated with sexual dysfunction [37]. In our previous study, sexual function slightly appreciated after treatment with *C. membranaceus* in tandem with a significant improvement in the quality of life (QoL). In another study, terazosin, an α-1 blocker, reduced cholesterol levels in BPH patients with high cholesterol values, while increasing HDL levels [38]. Long-term dutasteride therapy resulted in increased LDL levels [39]. Our previous experiments implicated an increase in lipids, such as HDL, after treatment with *C. membranaceus*. Additionally, Apo A was significantly reduced. Therefore, this appears to be the only study that has reported HDL reduction in BPH patients with phytotherapy.

In the literature, the roles of SphK1 and SphK2 remain controversial. Generally, SphK1 is thought to be pro-proliferative and SphK2 is pro-apoptotic. However, some studies have proposed the reverse. The levels of the individual sphingokinases did not show significant differences before and after treatment in this study, although a slight reduction of both occurred after treatment. The ratios of SphK1/SphK2 decreased significantly after treatment, signifying a greater drop in SphK1 compared to SphK2, and thus suggesting the dominance of SphK2 activity over that of SphK1. An increase in SphK1 appears to promote resistance in PCa chemotherapy [40]. We therefore postulate that the ratio of the two is a determining factor for the positive or negative growth of the prostate.

Reduced expression of SPL apparently favors cell growth. Silencing SphK1 expression in combination with increased SPL expression has been shown to decrease SIP content in prostate cancer cells, thus making them more sensitive to anticancer treatment. This combined strategy could be the future direction of cancer treatment [18].

Conversely, SPL increased significantly in this study. SPL is known for dephosphorilation of sphingosine-1-phosphate (SIP) and ceramide-1-phosphat, altering the rhostate balance toward apoptosis. Therefore, the increase in SPL after treatment signifies increased apoptosis, which aligns with previous in vitro and in vivo studies. SPL is the sole enzyme that irreversibly decreases the amount of intracellular SIP, by cleaving it into hexadecenal and ethanolamine phosphate, as the last steps in the sphingomyelin degradation pathway [41]. Other studies have demonstrated that an increase in SPL expression results from stress, irradiation [42], and chemotherapy [43,44] using non-cancerous cells.

The significant increase in ceramide obtained comparing before and after treatment values (*p* = 0.004) is the most convincing among the possible suggested pathways, meaning the ceramide pathway is involved in the phytotherapeutic action of *C. membranaceus*. Increasing the cytotoxic response to chemotherapy in prostate cells by increasing intracellular ceramide concentration may be worthwhile [45].

The degradation of ceramide involves ceramidases. The inhibition of ceramidase was thought to be a possible mechanism for increasing ceramide levels. A study using B13, an inhibitor of acid ceramidase, on human prostate cancer cell lines and xenografts showed that B13 produced tumors, and microscopic evaluation of the tumors demonstrated the presence of apoptosis [46]. In this study, the importance of the increase found in ceramide is corroborated by findings observed in other studies demonstrating that intracellular ceramide increases alone, and not a balance between SIP and ceramide, determines whether apoptosis will occur in LNCaP cells [47]. Furthermore, a strong correlation was observed in apoptosis, induced by serum deprivation in androgen-independent prostate cancer cell lines and ceramide in that study.

## 5. Conclusions

We conclude that *C. membranaceus* uses the ceramide pathway by modulating the SphK1/SphK2 ratio and the increase of SPL to generate oxidative stress, significantly increasing MDA, which supports the previously proven apoptotic effect, and PSA reduction activity, of the extract.

## Figures and Tables

**Table 1:** Parameters relating total PSA (TPSA) and free PSA (FPSA) shown as mean values before and after treatment.

Parameter (ng/mL)	Mean ± SD Before	Mean ± SD After	*p*-Value Before/After
TPSA	25.71 ± 20.10	16.15 ± 15.66	0.000 *
FPSA	4.54 ± 3.17	3.25 ± 2.57	0.018 *

* Signifies *p* < 0.05.

**Table 2:** Lipid profile including total cholesterol (TC), triglyceride (TG), high and low density lipoprotein (HDL, LDL), and Apolioprotein A-1 (APO A) and B, before and after treatment.

Parameter (mmol/L)	Mean ± SD Before	Mean ± SD After	*p*-Value Before/After
TC	5.03 ± 1.12	5.19 ± 1.15	0.423
TG	1.15 ± 0.43	1.22 ± 0.56	0.517
HDL	0.76 ± 0.33	0.88 ± 0.32	0.062
LDL	3.74 ± 1.03	3.75 ± 1.02	0.959
AP0 A	1.31 ± 0.45	1.52 ± 0.47	0.024 *
APO B	0.58 ± 0.37	0.50 ± 0.13	0.2221

* Signifies *p* < 0.05.

**Table 3:** Mean values of sphingosine phosphokinase 1 and 2 (SphK1 and SphK2) and their ratios, as well as sphingosine phosphate lyase (SPL), before and after treatment.

Parameter (ng/mL)	Mean ± SD Before	Mean ± SD After	*p*-Value Before/After
SPK 1	1.84 ± 1.34	1.50 ± 1.29	0.302
SPK 2	110.4 ± 57.67	98.63 ± 34.59	0.359
SPK1/SPK2	0.042 ± 0.018	0.017 ± 0.011	0.049 *
SPK Lyase	143.7 ± 24.20	161.4 ± 35.66	0.050 *

* Signifies *p* < 0.05.

**Table 4:** Mean ceramide levels before and after treatment.

Parameter (ng/mL)	Mean ± SD Before	Mean ± SD After	*p*-Value Before/After
Ceramide	64.77 ± 11.09	72.92 ± 10.83	0.004 *
MDA	41.15 = 16.00	52.95 = 15.18	0.047 *

* Signifies *p* < 0.05.

**Table 5:** A correlation between HDL and the individual analyses.

Parameter	r-Value Before	r-Value After	*p*-Value Before	*p*-Value After
TPSA (ng/mL)	−0.0741	0.2372	0.697	0.206
APO A (mmol/L)	0.2045	−0.2338	0.278	0.213
SPK2 (ng/mL)	0.0827	0.0936	0.664	0.622
SPK1 (ng/mL)	0.3826	−0.1649	0.036*	0.383
SPK Lyase (ng/mL)	0.0324	−0.2275	0.864	0.226
Ceramide (ng/mL)	0.4286	0.0227	0.018*	0.905

* Signifies *p* < 0.05.

**Table 6:** Correlation between SP-Lyase and lipids.

Parameter (mmol/L)	r-Value Before	r-Value After	*p*-Value Before	*p*-Value After
T. Chol	−0.1181	0.0634	0.5343	0.7393
TG	0.4370	0.0329	0.0158 *	0.8628
HDL	0.0324	−0.2275	0.8649	0.2266
LDL	−0.2229	0.1330	0.2365	0.4834

* Signifies *p* < 0.05.
